# Supplementary material for: Time from Symptom Onset to Diagnosis and Treatment among Haematological Malignancies: Influencing Factors and Associated Negative Outcomes
Source: Medicina (Kaunas). 2019 Jun 3;55(6):238. doi: 10.3390/medicina55060238 (PMC6631661; doi:10.3390/medicina55060238)

Supplementary Material

***Figure S1** Sociodemographic data collected from questioning participants*

1. Education: evaluated according to *The Education classification of Ministry of Education and Science of the Republic of Lithuania*
2. Employment before disease:
   ☐ Employed ☐ Unemployed due to:
   a. Unemployment
   b. Retirement
   c. Student
   d. Other____________
3. Marital status:
   ☐ Married ☐ Separated ☐ Widow ☐ Single ☐ Other___________
4. Anamnesis of oncological diseases among 1st order relatives:
   ☐ Yes: ☐ No
   1. Haematologic malignancies____
   2. Other oncologic diseases ____
5. Anxiety and depression: *evaluated according to HADS scale validated in Lithuania*

***Figure S2** Questions about symptom evaluation, reasons for delay and other patient-related factors (gathered while questioning patients)*

1. Did you have any symptoms (connected with haematologic malignancy)?
   ☐ YES: ☐ NO (skip to question **14**)
   1. Temperature >38°C
   2. Weight loss:
      a. How many kilograms per specific time interval?_____________
   3. Night sweats
   4. Fatigue
   5. Lymphadenopathy
   6. Pruritus
   7. Discomfort in abdomen
   8. Easily occurring bleeding or bruises
   9. Pain in bones
   10. Other ____
2. When did you noticed first disease symptoms?
3. When did you first registered for a medical consultation due to these symptoms?
4. When was the first medical consultation?
11. Sometimes people put off going to see the doctor, could you say if any of these stop you from going to see the doctor?
    1. I was too embarrassed: ☐ YES ☐ NO
    2. I was too scared: ☐ YES ☐ NO
    3. I was worried about wasting the doctor's time: ☐ YES ☐ NO
    4. My doctor is difficult to talk to: ☐ YES ☐ NO
    5. It is difficult to make an appointment with my doctor: ☐ YES ☐ NO
    6. I was too busy to make an appointment with my doctor: ☐ YES ☐ NO
    7. I had too many other things to worry about: ☐ YES ☐ NO
    8. It was difficult for me to arrange transport to see the doctor: ☐ YES ☐ NO
    9. I was worried about what the doctor might find: ☐ YES ☐ NO
    10. I didn't feel confident talking about my symptoms with the doctor: ☐ YES ☐ NO
    11. I didn't have somebody to accompany me for a doctor visit: ☐ YES ☐ NO
    12. I thought that symptoms will disappear on their own: ☐ YES ☐ NO
    13. I thought that symptoms are occurring because of other disease (for example fatigue due to cold): ☐ YES __________ ☐ NO
    14. I thought that symptoms are due to non-medical condition (for example fatigue due to stress): ☐ YES __________ ☐ NO
    15. Other ☐ YES_________ ☐ NO
12. What do you think had the biggest impact on delaying registration for a doctor visit?
13. What do you think had the biggest influence on the time interval from registration to a first medical consultation?
14. What do you think had the biggest influence on the time interval between first medical consultation and correct diagnosis?
15. ~~What do you think had the biggest impact on the time interval between correct diagnosis and treatment initiation?~~
16. Did you use any medication on your own in order to treat your symptoms before correct diagnosis? ☐ YES __________ ☐ NO

Figure S3 *Questions about health system related factors (gathered while questioning patients)*

17. Which doctor you saw first due to haematologic malignancy symptoms? (if patient chose 2, 3, 4 or answer 5 go to question 19)
    1. General practitioner
    2. Emergency department doctor
    3. Specialist, but not haematologist___________________________
    4. Haematologist
    5. Other____
18. Which specialist general practitioner referred you in the first place?
    1. Haematologist
    2. A few specialists – haematologist and another specialty doctor _____________________
    3. Non haematologist ______________________________________
19. Diagnosis suspected after first medical consultation?
    1. Oncologic diagnosis
    2. Other, non-malignant disease (for example infection)
    3. Symptoms administered to other disease diagnosed previously
    4. Other____
20. Did the first doctor you consulted prescribe medicine for your condition?
    ☐ YES: ☐ NO
    1. Symptomatic treatment
    2. Treatment for another, non-malignant, disease
    3. Other____
21. How many specialists you visited before correct diagnosis?

Figure S4 *Data gathered from medical records*

1. When was haematologic malignancy diagnosis confirmed? ____
2. When was haematologic malignancy treatment started? ____
3. Disease type: ☐ LYMPHOMA: ☐ MULTIPLE MYELOMA:
   - LYMPHOMA:
     1. Hodgkin:
        Ann-Arbor stage __________
     2. Non-Hodgkin: aggressive or indolent (underline):
        Ann-Arbor stage ________
        Exact type _________________
   - MULTIPLE MYELOMA:
     1. ISS stage __________________
     2. Durie-Salmon stage__________
4. Multiple myeloma complications at the time of diagnosis:
   ☐ YES: ☐ NO
   1. Hypercalcemia – serum calcium >0,25mmol/l (>1mg/dl) over upper normal value limit or >2,75mmol/l (>11mg/dl)
   2. Kidney insufficiency – creatinine clearance <40ml per min, serum creatinine >177mikromol/l (>2mg/dl)
   3. Bone lesions – one or more osteolytic bone lesion in rentgenography, CT, PET-CT
   4. Anemia – haemoglobin >20 g/l lower than normal value or < 100g/l
   5. Infectious diseases – 2 or more bacterial infections in 12 months
5. Gender: ☐ Male ☐ Female
6. Age at the time of diagnosis: __________
7. Place of residence: ☐ City ☐ Smaller village
   1. The biggest cities in Lithuania – Vilnius, Kaunas
   2. Other cities_______________
8. CIRS value: ________

Figure S5 *Lymphomas distribution according to disease stage (a) and NHL type (b).*

HL – Hodgkin lymphoma; NHL – non Hodgkin lymphoma

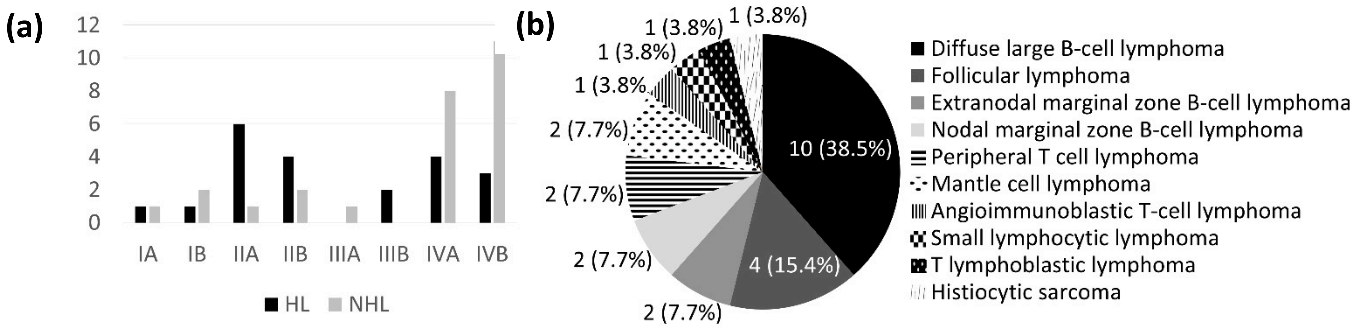

Figure S6 *The most important reasons for delay in A, B, C, D intervals according to participants*

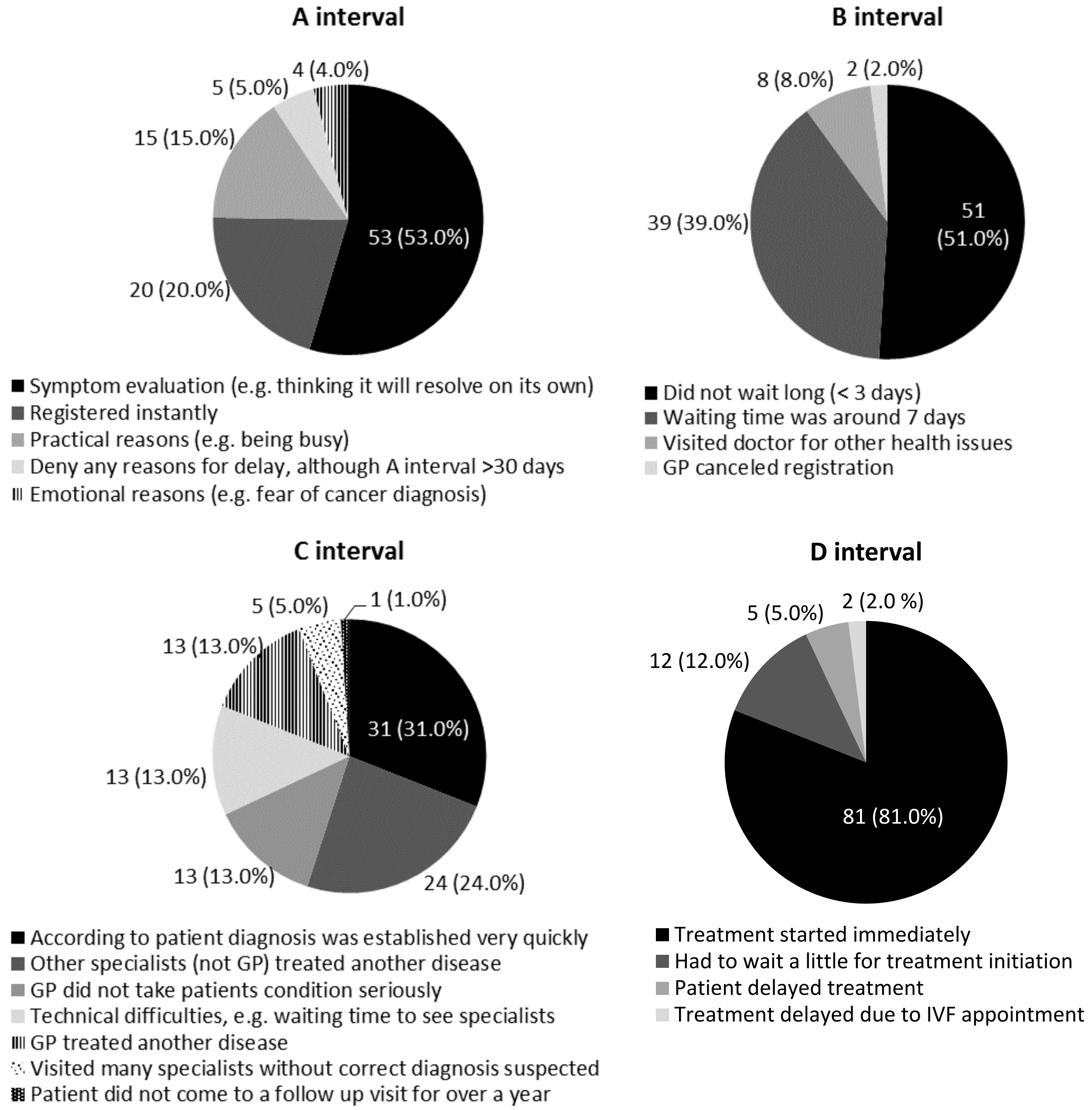

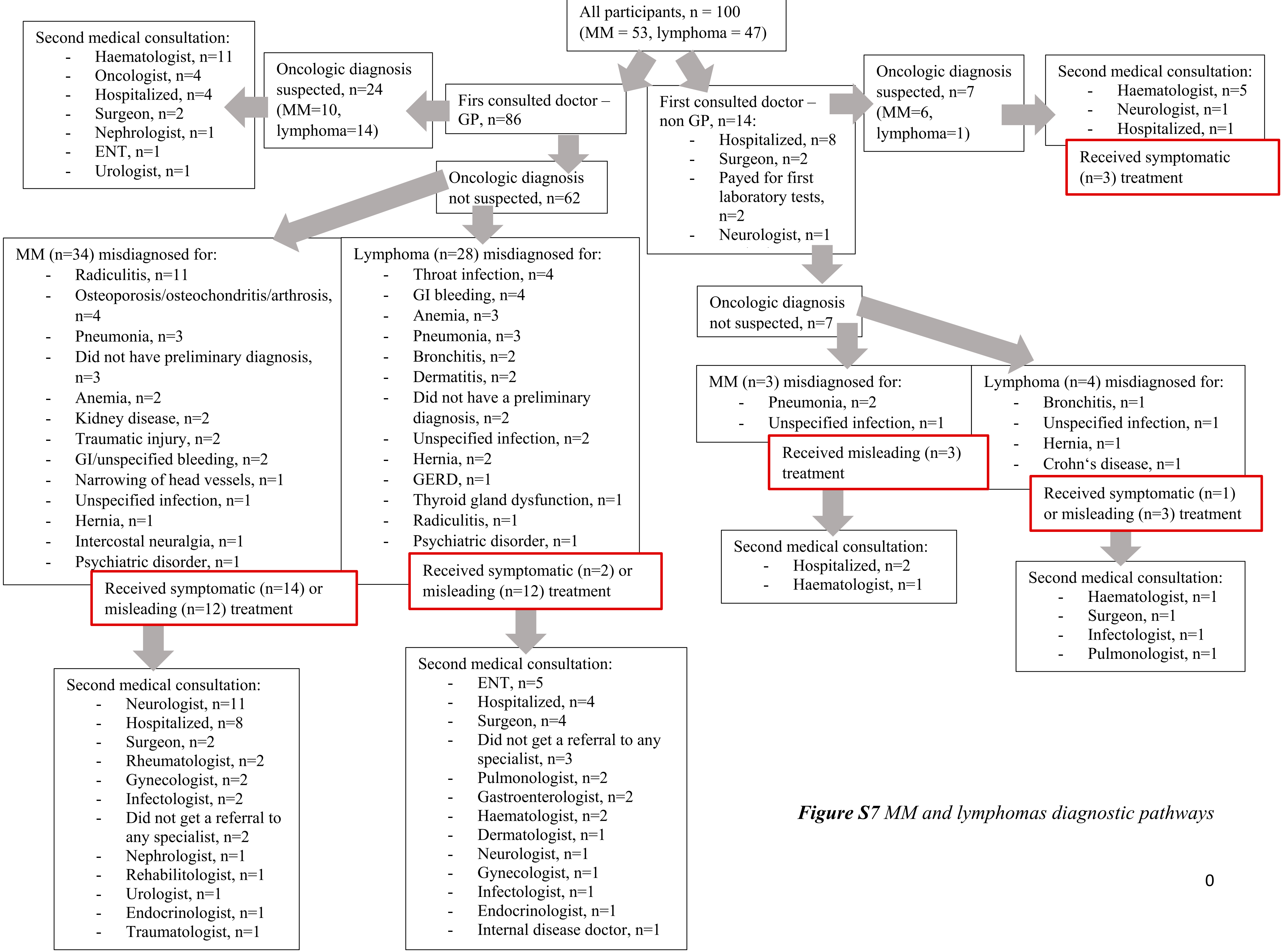

Figure S7 *MM and lymphomas diagnostic pathways*

Figure S8 *Number of patients for 1 PCP (primary care physician – including GPs and general pediatricians), in Lithuanian counties and biggest cities*

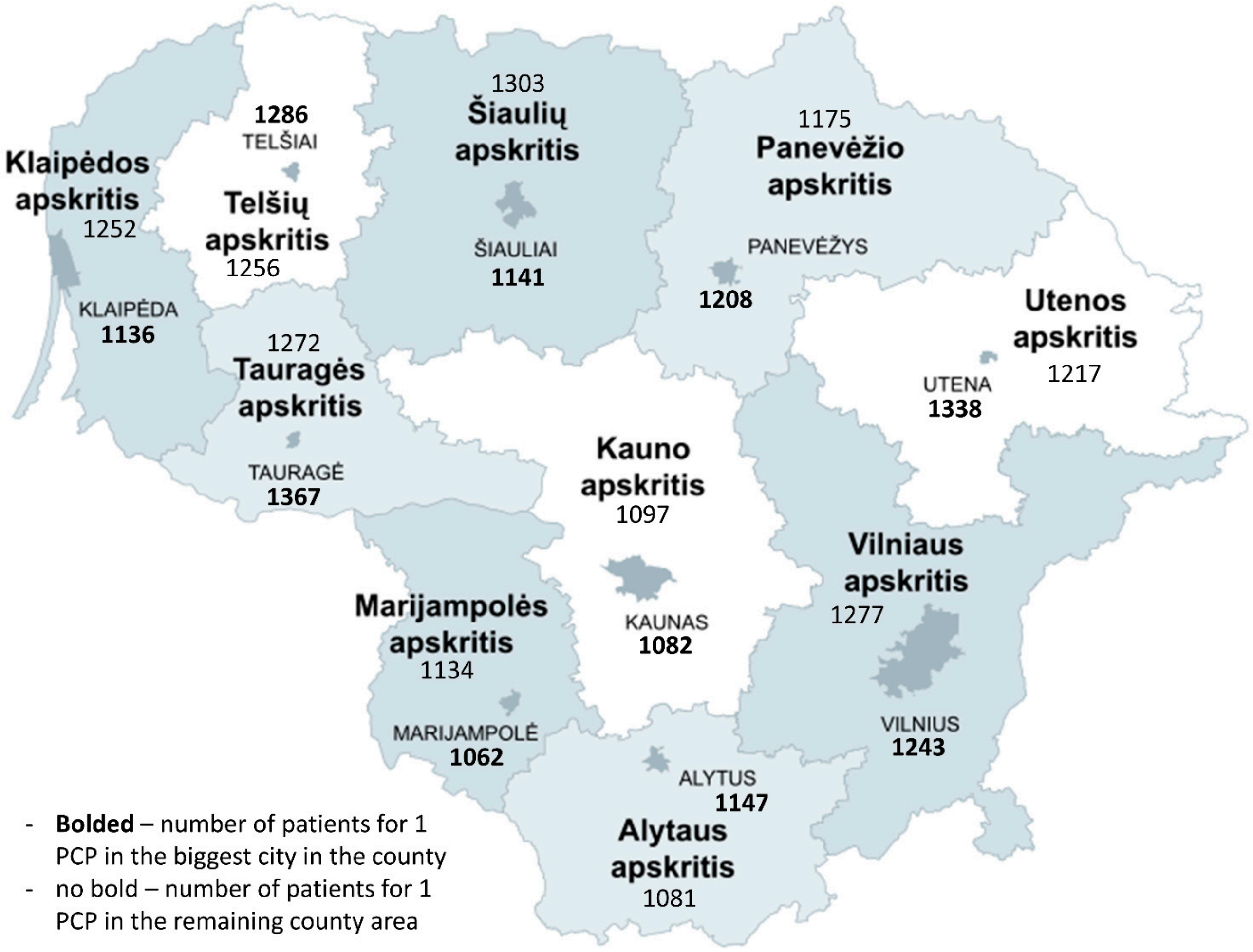

Supplement: Supplementary file 1 [file medicina-55-00238-s001.pdf]
